# ASL reveals regional brain perfusion impairment in neonates with mild hypoxic ischemic encephalopathy

**DOI:** 10.1038/s41598-025-17246-0

**Published:** 2025-08-28

**Authors:** Mario Cirillo, Simona Puzone, Alessandro Pasquale De Rosa, Lorenzo Ugga, Elisabetta Caredda, Mario Diplomatico, Massimiliano De Vivo, Maria Agnese Pirozzi, Alessandra D’Amico, Emanuele Miraglia Del Giudice, Fabrizio Esposito, Paolo Montaldo

**Affiliations:** 1 Department of Advanced Medical and Surgical Sciences, Advanced MRI Research Center, University of Campania “Luigi Vanvitelli”, Naples, Italy; 2 Department of Woman, Child, and General and Specialized Surgery, University of Campania “Luigi Vanvitelli”, Naples, Italy; 3 Department of Neonatal Intensive Care, AORN Sant’Anna e San Sebastiano, Caserta, Italy; 4 Department of Neonatal Intensive Care, AORN San Giuseppe Moscati, Avellino, Italy; 5 Department of Neonatal Intensive Care, Monaldi Hospital, Naples, Italy; 6 Department of Radiology, “Tortorella” Private Hospital, Salerno, Italy; 7 Centre for Perinatal Neuroscience, Department of Brain Sciences, Imperial College London, 5th floor Hammersmith House Du Cane Road, London, W12 0HS UK

**Keywords:** Arterial spin labelling, Magnetic resonance imaging, Biomarkers, Neonatal encephalopathy, Perfusion, Neuroscience, Neurology, Predictive markers

## Abstract

There is a lack of neuroimaging data and effective biomarkers in infants with mild hypoxic ischaemic encephalopathy (HIE). Cerebral reperfusion injury has shown potential as marker of neurodevelopmental outcome in moderate and severe HIE. We examined cerebral perfusion by using arterial spin labelling (ASL) in infants with mild HIE and its associations with adverse outcomes. We also studied the presence of any potential regional sensitivity of cerebral blood flow (CBF) on the effects of HIE severity. This prospective cohort study included term and near-term neonates admitted for HIE across 3 neonatal intensive care units in Italy between October 2019–2022. Magnetic resonance imaging (MRI)-ASL was performed between 4 and 10 days after birth. Neurodevelopmental outcome was assessed at 24–28 months. Of the 94 infants included in the analysis, 74 neonates had mild [79%], 15 moderate [16%], 5 severe encephalopathy [5%]. Of the 71 neonates with mild HIE and neurodevelopmental outcome available, 15 (21%) showed mild disability. Basal ganglia CBF was the only region significantly associated with cognitive, motor and language Bayley scores (false-discovery-rate < 0.05). HIE severity had a regional dependent effect with involvement of Heschl, Rolandic operculum, limbic lobe, subcortical gray nuclei followed by frontal lobes. Basal ganglia CBF in infants with mild HIE was associated with adverse outcomes even without any MRI visible deep brain nuclei injury.

**Supplementary Information:**

The online version contains supplementary material available at 10.1038/s41598-025-17246-0.

## Introduction

Neonatal hypoxic ischaemic encephalopathy (HIE) is a major cause of neonatal mortality and long-term disability worldwide occurring in 3–5 per 1000 live birth^1^.

Neonates with mild HIE have been considered for years a low-risk population as they were thought to have favourable neurodevelopmental outcomes without any long-term disability. However, recent evidence has demonstrated that a significant proportion of these neonates develop long-term neuro-developmental impairment, including learning and neuropsychological difficulties, autism, visual and sensory loss^2–4^. Furthermore, recent studies suggest that up to 61% of neonates with mild HIE have abnormal brain magnetic resonance imaging (MRI), a similar proportion compared with the moderate, severe HIE population^5^.

In the biochemical cascade following hypoxic ischemic injury, the restoration of brain perfusion (“reperfusion injury”) is the primary target for neuroprotective interventions in moderate and severe HIE. Arterial spin labelling (ASL) is a non-invasive perfusion MRI method without contrast material administration or radiation, which may help to assess regional cerebral perfusion^6^. Different studies have examined the use of ASL in neonates with HIE. This evidence demonstrated high accuracy of this MRI technique thus suggesting its potential as surrogate biomarker and its utility in evaluating neuroprotective therapies^7–11^. However, none of these studies assessed whether there was any association between cerebral blood flow (CBF) measured by ASL and long-term outcome in mild HIE.

In these neonates, the timing of cerebral injury remains uncertain, as it is unclear whether the brain injury observed occurred prior to birth, thus allowing the infant an opportunity for partial recovery by the time of delivery. A better understanding of the features of brain injury in this population may help to promptly identify the neonates who are at higher risk of adverse outcomes, thus informing future neuroprotection studies.

The purpose of this study was to investigate whether cerebral perfusion assessed with ASL was related to the neurological outcomes at 2 years after mild HIE. We also studied the presence of any potential regional sensitivity of cerebral perfusion on the effects of HIE severity.

## Materials and methods

### Study design and participants

This prospective multicentre study included all consecutive neonates admitted for HIE to three neonatal intensive care units, all part of the University of Campania “Luigi Vanvitelli” network, between October 2019 and October 2022. The study was approved by the University of Campania “Luigi Vanvitelli” ethics committee and all methods were performed in accordance with relevant guidelines and regulations. Written informed consent was obtained from parents or legal guardian(s). All neonates with birth weight > 1800 g and gestational age > 35 weeks with evidence of recent intrapartum hypoxia-ischemia (10-minute Apgar score < 6; continued need for resuscitation, including endotracheal or mask ventilation, at 10 min after birth; and/or birth acidosis, defined as pH of < 7.0 or base excess of > 16 mmol/L in any cord or neonate gas sample within 60 min of birth) were eligible. Additional evidence of perinatal asphyxia, such as an acute obstetric event (e.g. late or variable decelerations, cord prolapse, cord rupture, uterine rupture, maternal trauma, hemorrhage, or cardiorespiratory arrest) was required if the pH or base excess was < 7.15 to 7.0 and/or 10–16 mmol/L in the umbilical cord.

Neurological examination was performed by certified examiners using a standardised neurological examination. Mild HIE was defined as 2 or more abnormal categories (consciousness level, spontaneous activity, posture, tone, primitive reflexes, and autonomic nervous system) but not meeting the diagnosis of moderate or severe HIE (*≥* 3 moderate or severe abnormalities). Attending clinician decided whether to offer therapeutic hypothermia to neonates with mild HIE. Neonates without HIE of any grade or life-threatening congenital malformation were excluded.

MRI was centrally performed on a 3.0 Tesla scanner (GE HealthCare) equipped with a 32-channel head coil between 4 and 10 days after birth. Spontaneously breathing infants were sedated with a single dose of intranasal dexmedetomidine (3 mcg/kg) and intranasal midazolam (0.2 mg/kg). The MRI protocol included 2-dimensional axial T2weighted (T2w) (spatial resolution = 0.33 × 0.33 × 2 mm, repetition time = 7000 ms, echo time = 117 ms, flip angle = 142°, matrix size = 512 × 512) and 3-dimensional pseudo-continuous arterial spin labeling (PCASL) (spatial resolution = 1.56 × 1.56 × 4 mm, repetition time = 4722 ms, echo time = 11 ms, flip angle = 111°, labeling duration = 1450 ms, post-label delay = 2025 ms, matrix size = 128 × 128) sequences. Each PCASL sequence reconstructed the difference (control minus labeled) image and the perfusion calibration image (M0). A neuroradiologist with more than 10 years of experience in neonatal neuroimaging reviewed brain MR images of all neonates by using a validated scoring system^12^.

Adverse outcome was defined as death or mild or moderate or severe disability in survivors at 24–28 months of age. Neurodevelopmental assessments were performed by using the Bayley Scales of Infant and Toddler Development III Edition which provide cognitive, language, and motor composite scores. The presence of disability was determined according to the NICHD Neonatal Research Network: (a) mild: cognitive score of 70–84 alone, or a cognitive score ≥ 85 and GMFCS level 1 or 2, seizure disorder (without anti-epileptic medication), or hearing deficit with ability to follow commands without amplification; (b) moderate: cognitive score from 70 to 84 and GMFCS level 2, active seizure disorder (receiving anti-epileptic medication), or hearing deficit with the ability to follow commands after amplification; and (c) severe: cognitive score < 70, GMFCS level 3–5, blindness, or hearing impairment with inability to follow commands in spite of amplification^13,14^.

### Quantitative ASL analysis

For delineation of regions of interest (ROIs), the University of North Carolina Infant Atlas for neonates was used^15^,which comprises the Automated Anatomical Labelling (AAL) parcellation map^16^. MRI pre-processing steps included T2w skull stripping with Brain Extraction Tool^17^, T2w co-registration to the M0 reference image with FLIRT^18^ and atlas inverse warping into M0 space with Advanced Normalization Tools (ANTs)^19^. The output of each pre-processing step was visually inspected to verify the correctness of the registration. The perfusion weighted images were also visually inspected (blinded to the clinical details) for any motion artifacts and in case of severe artifacts the subjects were excluded.

**Fig. 1:**
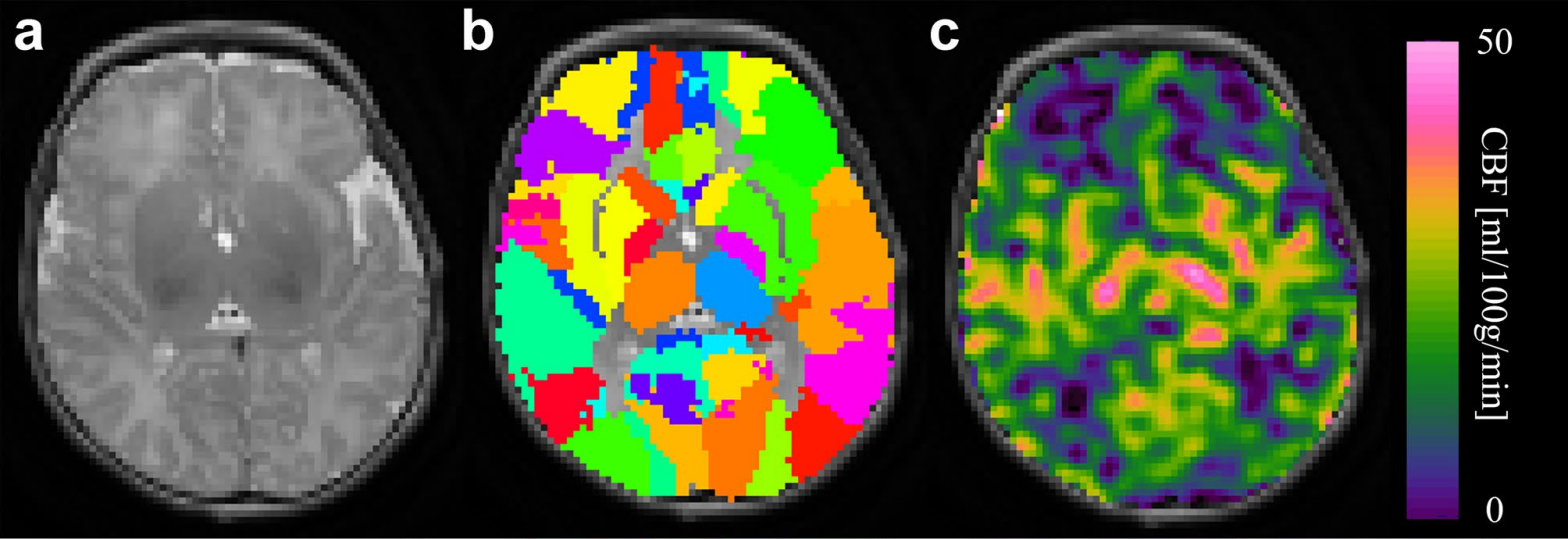
Axial views of a T2W image (**a**), ROI as overlay (**b**) and CBF map (**c**).

Cerebral blood flow (CBF) estimation was performed with the BASIL tool^20^. Quantitative CBF maps, calibrated in physical units (mL/100 g/min), were obtained by applying a single-compartment kinetic model inversion to the PCASL difference image, with voxel-wise calibration using the M0 image^21^. Regional CBF values were then extracted from 90 ROIs (45 for each hemisphere) of the AAL infant parcellation (**Supplementary Table 1**) (Fig. 1). No statistically significant difference was observed between left and right hemisphere in CBF. Therefore, the CBF value of each ROI was averaged between the two hemispheres.

### Statistical analysis

Data were analysed using IBM SPSS version 29 (IBM Corp) and R, version 4.4.1 (R Project for Statistical Computing). The level of significance was set at *P* < 0.05. We described quantitative variables by using median or mean value and interquartile range [IQR] or standard deviation (SD), according to their distribution. Continuous data were assessed for normality by visual inspection of histograms and Q-Q plots. Bivariate analysis was performed using Pearsonʼs chi-squared test or Fisherʼs exact test for categorical data, and Studentʼs t-test or Mann–Whitney U test for continuous variables. The relationship between the severity of injury on conventional MRI and the HIE severity stage was assessed by linear-by-linear association.

In order to assess the effects of the outcome at 2 years (normal vs. adverse outcome) on the overall cerebral perfusion in the infants with mild HIE we used an analysis of covariance (ANCOVA). In this model the dependent variable was the CBF averaged across all the ROIs. We then tested the presence of a relationship between outcome at 2 years in infants with mild HIE and the CBF values in each of the different ROIs using logistic regression with a backward stepwise conditional method. A multiple linear regression model was used to examine the joint association between all the CBF values and the cognitive, language, and motor composite scores obtained with Bayley-III assessments. We adjusted all the regression models for birth gestational age, gender, postnatal age at MRI, haematocrit values^22^, need for inotropic support and therapeutic hypothermia.

We performed ANCOVA to assess the linear dependence of the whole cerebral perfusion values (CBF averaged across all the ROIs) with HIE severity stage (mild vs. moderate/severe) as a between-group factor. Finally, to examine these effects across all ROIs simultaneously while controlling for their mutual dependence, we used a multivariate analysis of variance (MANOVA). These analyses were adjusted for birth gestational age, gender, postnatal age at MRI, haematocrit values^22^, need for inotropic support and therapeutic hypothermia. A false-discovery-rate (FDR) correction for multiple comparisons was applied with FDR < 0.05 considered as statistically significant.

## Results

### Study population

**Fig. 2:**
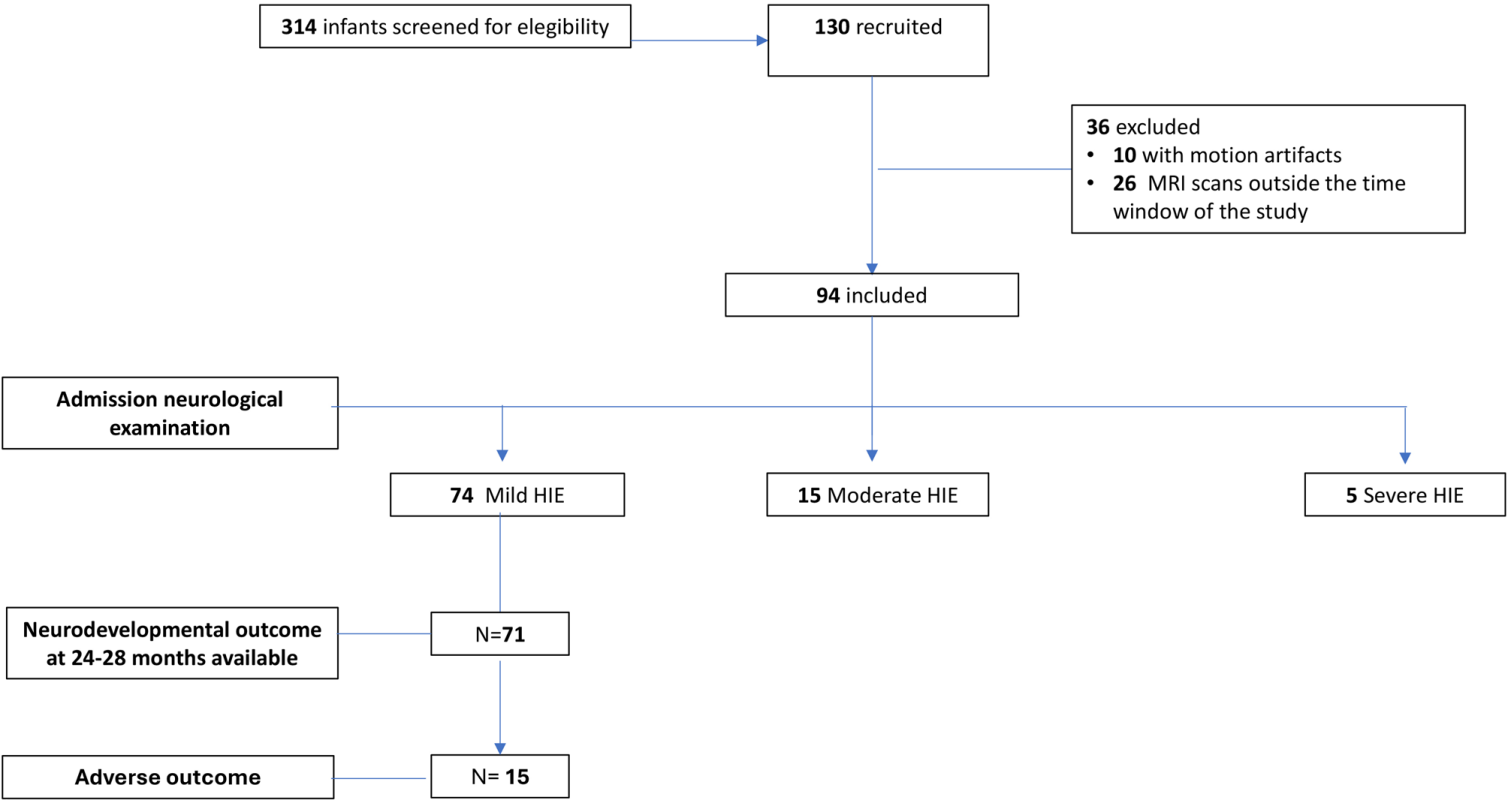
Flow-chart of the study.

Of the total 130 neonates with HIE recruited into the study, 94 (72%) were included in the analysis (Fig. 2). Seventy-four neonates showed mild [79%], 15 moderate [16%], 5 severe encephalopathy [5%]. Mean (SD) gestational age and birth weight were 39 (1.53) weeks and 3202 (508) grams. Conventional MRI was performed at a mean (SD) postnatal age of 6.9 (1.7) days. There was no difference in the CBF values in cortical and deep gray matter structures in cooled and non-cooled infants with mild encephalopathy (**Supplementary Table 2**). Baseline characteristics are shown in Table 1.

**Table 1:** Demographic characteristics among infants with hypoxic ischemic encephalopathy.

Characteristics^a^	Mild HIE (*N* = 74)	Moderate HIE (*N* = 15)	Severe HIE (*N* = 5)	*P* value^b^
Gestational age, weeks	39.7 (1.5)	39.9 (1.6)	37.8 (3.2)	0.20
Birth weight, g	3394 (390)	3291 (281)	3141 (856)	0.06
Female participants	25 (34)	6 (40)	2 (40)	0.81
Cord arterial blood pH	7.02 (0.09)	6.98 (0.13)	6.87 (0.16)	0.01
Apgar 1 min	4 [3–5]	2 [1–3]	2 [0–5]	0.005
Apgar 5 min	7 [6–8]	5 [5–6]	5 [1–6]	0.001
Apgar 10 min	8 [7–9]	7 [6–8]	6 [4–8]	0.006
Instrumental Delivery	13 (17.5)	4 (26.6)	0 (0)	0.35
Emergency CS	21 (28.3)	6 (40)	3 (60)	0.23
Sentinel events^c^	21 (28.3)	1 (6.6)	1 (20)	0.43
CTG abnormalities	24 (32.4)	2 (13.3)	0 (0)	0.25
Meconium staining	21 (28.3)	4 (26.6)	2 (40)	0.92
Therapeutic hypothermia	44 (59.4)	15 (100)	5 (100)	0.003

*HIE*, Hypoxic ischemic encephalopathy, *CTG*, Cardiotocography, *CS*, Caesarean Section.

^a^ Data are presented as a number (percentage) for nominal variables and mean (standard deviation) or median [IQR] for continuous variables. ^b^ P values for the differences of nominal and continuous variables among the three groups were determined by Chi-square test and analysis of variance (ANOVA) test, respectively. ^c^ Sentinel events were defined as cord mishap, lengthened second stage, obstructed labor, shoulder dystocia, antepartum haemorrhage, uterine rupture.

### Brain perfusion and neurological outcome in mild HIE

Among the 74 children who had mild HIE, 3 were lost to follow-up. Neurodevelopmental assessments were performed at a mean age of 26 (SD 1.5) months. Fifty-six out of 71(79%) infants demonstrated a normal outcome while 15 (21%) showed mild disability (Table 2). None of the patients showed cerebral palsy.

**Table 2:** Comparison of baseline characteristics and Bayley III scores in neonates with mild hypoxic ischemic encephalopathy according to their outcomes.

Characteristics^a^	Good Outcome (*N* = 56)	Adverse Outcome (*N* = 15)	*P* value^b^
Gestational age, weeks	39.4 (1.51)	39.26 (1.92)	0.79
Birth weight, g	3413 (352)	3151 (529)	0.02
Female participants	21 (37)	4 (27)	0.54
Cord arterial blood pH	7.02 (0.09)	7.04 (0.08)	0.27
Apgar 1 min	4 [2–5]	5 [2–6]	0.46
Apgar 5 min	7 [6–8]	6 [5–7]	0.80
Apgar 10 min	8 [7–9]	8 [6–9]	0.93
Delivery			
Instrumental Delivery	10 (18)	3 (20)	1
Emergency CS	17 (30)	1 (7)	0.09
Sentinel events^c^	17 (30)	4 (27)	0.92
CTG abnormalities	19 (34)	4 (27)	0.48
Meconium staining	16 (28)	4 (27)	0.95
Therapeutic hypothermia	29 (52)	11 (73)	0.23
Neurodevelopmental outcome at 24–28 months			
Cognitive composite score	102.1 (8.4)	79.3 (7.9)	< 0.001
Language composite score	98.9 (11.2)	76.8 (15.5)	< 0.001
Motor composite score	103.5 (8.1)	84.2 (9.1)	< 0.001
Cognitive composite score < 85	-	15 (100)	< 0.001
Language composite score < 85	5 (9)	8 (53)	< 0.001
Motor composite score < 85	1 (2)	8 (53)	< 0.001

*CS*, Caesarean section.

^a^Data are presented as a number (percentage) for nominal variables and mean (standard deviation) or median [IQR] for continuous variables. ^b^P values for the differences of nominal and continuous variables between the two groups were determined by the Chi-square test and the Student’s t- test, respectively.^c^ Sentinel events were defined as cord mishap, lengthened second stage, obstructed labor, shoulder dystocia, antepartum haemorrhage, uterine rupture.

The effect of the outcome on the overall cerebral perfusion was analysed with ANCOVA while controlling the effect for birth gestational age, gender, postnatal age at MRI, haematocrit values and therapeutic hypothermia. The analysis showed that infants with adverse outcome showed a significantly higher whole brain CBF than those with good outcome (*P* = 0.02). This difference remained significant when each of the different regions was analysed separately (**Supplementary Table 3**). Multivariable logistic regression with a backward stepwise conditional regression analysis demonstrated that mean basal ganglia (putamen) was an independent predictive factor for neurodevelopmental outcome at 2 years (odds ratio = 1.59, 95% CI, 1.22–2.08; *P* = 0.001).

**Fig. 3:**
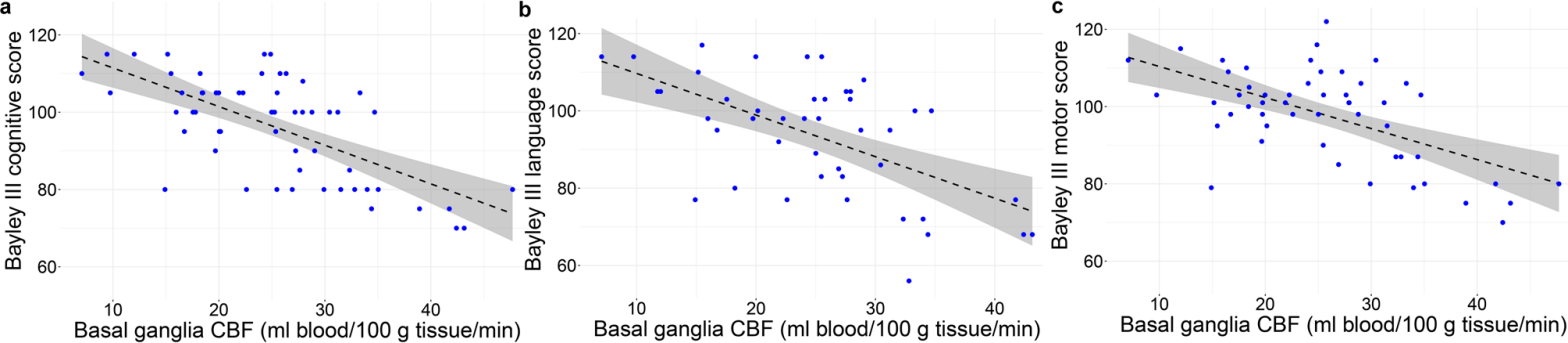
Relation of basal ganglia (putamen) cerebral blood flow with Bayley-III cognitive (**a**), language (**b**), and motor (**c**) scores at follow-up. Each blue dot is a patient and the black dotted line represents the regression line. The grey shaded area represents the 95% confidence interval.

We then examined the relationship between CBF measures and the continuous neurodevelopmental outcome scores from Bayley III assessment. In the multivariable analysis, basal ganglia (putamen) CBF was the only region significantly associated with all three scores (FDR = 0.02, β coefficient − 1.23, 95% CI, -3.75, -0.44 cognitive score; FDR = 0.01, β coefficient − 1.33, 95% CI, -4.73, − 0.70 language score; FDR = 0.03 β coefficient − 1.46, 95% CI, -3.73, − 0.62 motor score) (Fig. 3). We observed a negative association between Bayley III scores and basal ganglia (putamen) CBF. All the other ROIs did not show any significant associations with Bayley scores (FDR > 0.05).

### Brain perfusion according to HIE severity

Only 2/74 (3%) infants with mild HIE showed brain injury to the basal ganglia area whereas 29/74 (39%) showed white matter injury (Table 3). The median age at the time of MRI was 7 (minimum 4, maximum 9, IQR 2.6) days in infants with brain injury (basal ganglia and/or white matter injury) and 6.2 days (minimum 4, maximum 9, IQR 2.8) (*p* = 0.42) in infants without it. Mean (SD) basal ganglia (putamen) CBF was significantly higher in infants with mild HIE and brain injury (basal ganglia and/or white matter injury) compared with those without it (32.90 (15.90) versus 25.35 (8.68) ml/100 g tissue/min respectively, *p* = 0.03).

**Table 3:** Brain injury on conventional MRI according to the severity of hypoxic ischemic encephalopathy.

Area^a^	Grade	Mild HIE (*N* = 74)	Moderate HIE (*N* = 15)	Severe HIE (*N* = 5)	*P* value^b^
Basal ganglia and thalami	0	72 (97.3)	11 (73.3)	1 (20)	< 0.001
	1	1 (1.3)	1 (6.7)	4 (80)	
	2	1 (1.3)	3 (20)	0	
PLIC	0	74 (100)	14 (93.3)	0	< 0.001
	1	0	1 (6.7)	0	
	2	0	0	2 (40)	
	3	0	0	3 (60)	
White Matter	0	45 (61)	10 (66.7)	0	0.006
	1	13 (17.6)	2 (13.3)	4 (80)	
	2	16 (22)	3 (20)	1 (20)	
Cortex	0	66 (89.2)	13 (86.6)	1 (20)	< 0.001
	1	7 (9.5)	1 (6.7)	3 (60)	
	2	1 (1.3)	1 (6.7)	1 (20)	

*MRI*, Magnetic resonance imaging, *HIE*, Hypoxic ischemic encephalopathy.

^a^ Data are presented as a number (percentage); ^b^ P values for the differences of ordinal variables between the three groups was determined by linear-by-linear association.

**Fig. 4:**
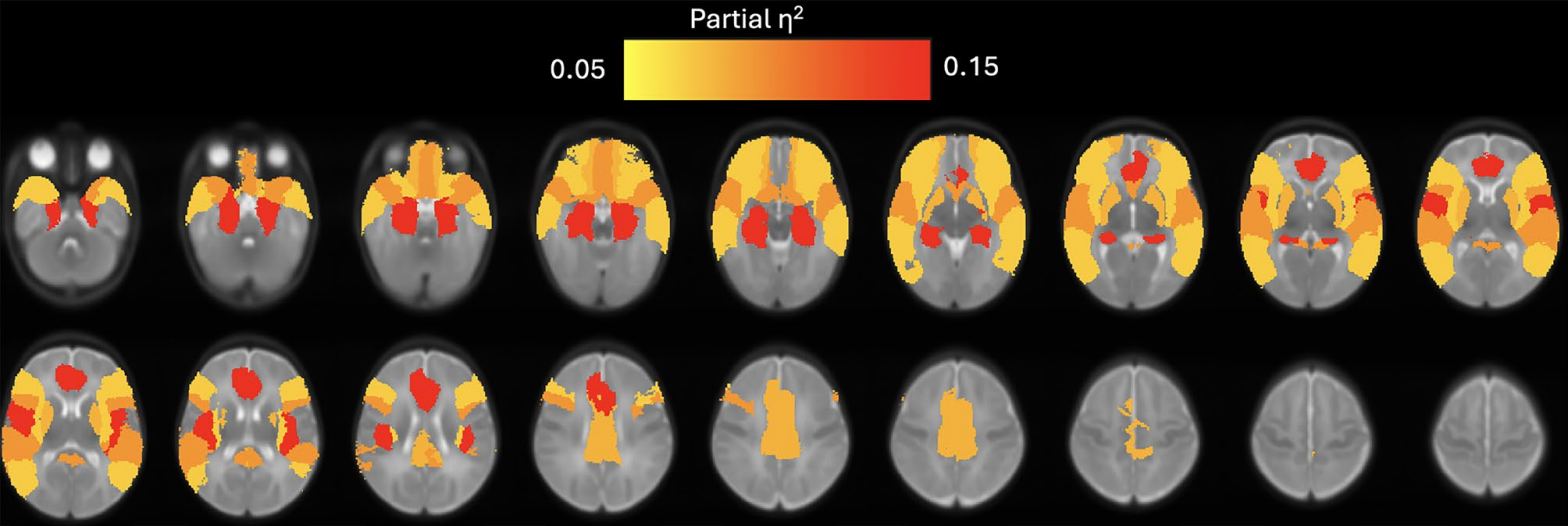
Brain map showing the statistically significant cortical and subcortical regions highlighted. The colour is based on the effect size (partial η2 value) for each of the significant regions identified by the multivariate analysis of variance (MANOVA).

Infants with moderate or severe HIE had a significantly higher whole brain CBF than those with mild HIE (*F* (1, 79) = 3.86; *P* = 0.04; partial η^2^ = 0.05). After controlling for birth gestational age, gender, postnatal age at MRI, haematocrit values, need for inotropic support and therapeutic hypothermia, MANOVA demonstrated that HIE severity had a statistically significant effect on the CBF values of the Heschl gyrus, Rolandic operculum, limbic lobe, subcortical gray nuclei followed by frontal lobes **(**Table 4**)**. The statistically significant ROIs are illustrated in Fig. 4 and **Supplementary Fig. 1**.

**Table 4:** Cortical and deep Gray matter structures whose perfusion was associated with the severity of hypoxic ischemic encephalopathy.

Regions of interest	Partial η2	FDR^a^	F
Central region			
*Rolandic operculum*	0.18	0.03	8.04 (1, 72)
Frontal lobe			
Lateral surface			
*Inferior Frontal gyrus*,* orbital part*	0.07	0.04	5.87 (1, 72)
*Inferior Frontal gyrus*,* opercular part*	0.09	0.03	7.18 (1, 72)
*Inferior Frontal gyrus*,* triangular part*	0.07	0.04	5.88 (1, 72)
Orbital surface			
*Superior frontal gyrus*,* medial orbital*	0.08	0.04	6.32 (1, 72)
*Middle Frontal gyrus*,* orbital part*	0.07	0.04	4.62 (1, 72)
*Gyrus rectus*	0.09	0.03	7.42 (1, 72)
*Olfactory cortex*	0.09	0.03	7.20 (1, 72)
Temporal lobe			
Lateral surface			
*Superior temporal gyrus*	0.09	0.03	7.17 (1, 72)
*Heschl gyrus*	0.09	0.03	7.52 (1, 72)
*Middle temporal gyrus*	0.07	0.04	6.06 (1, 72)
Limbic lobe			
*Temporal superior pole*	0.09	0.03	7.83 (1, 72)
*Temporal middle pole*	0.07	0.04	5.49 (1, 72)
*Anterior cingulate cortex and paracingulate gyri*	0.19	0.03	9.12 (1, 72)
*Median cingulate cortex and paracingulate gyri*	0.08	0.04	6.72 (1, 72)
*Posterior cingulate cortex*	0.09	0.03	7.91 (1, 72)
*Hippocampus*	0.18	0.03	8.20 (1, 72)
*Parahippocampal gyrus*	0.21	0.03	10.25 (1, 72)
*Insula*	0.07	0.04	6.10 (1, 72)
Subcortical gray nuclei			
*Amygdala*	0.17	0.03	8.12 (1, 72)
*Lenticular nucleus*,* Putamen*	0.08	0.04	6.34 (1, 72)

*FDR*, false discovery rate, MANOVA multivariate analysis of variance.

^a^The MANOVA was adjusted for birth gestational age, gender, postnatal age at MRI, haematocrit values, need for inotropic support and therapeutic hypothermia. A false-discovery-rate correction for multiple comparisons was applied.

## Discussion

At present neonates with mild HIE are increasingly recognised at high risk of adverse outcomes including brain injury and neurodevelopmental impairment. Therefore, there is a pressing need for the development and validation of prediction biomarkers in this population. In this study we showed that CBF assessed with ASL was significantly higher in the basal ganglia of infants with mild HIE who later developed adverse outcomes, even though only a minority of them showed basal ganglia thalami injury on conventional MRI. Furthermore, perfusion in the basal ganglia strongly correlated with cognitive, language and motor scores at 2 years of age, with higher CBF values associated with lower Bayley scores.

Neonatal abnormal brain perfusion plays a critical role in brain injury as it reflects metabolic demand and neuronal function. Following the hypoxic-ischemic insult, there is an initial reduction in CBF, followed by reperfusion and release of inflammatory molecules, leading to “reperfusion injury”, which is indicative of impaired autoregulation and usually peaks in the first 3 days of age. While some research showed gradual lowering of the CBF after the first 3 days of age^23,24^, other longitudinal studies demonstrated that hyperperfusion can be still detected later^9,10^ especially when white matter injury occurs^25^. This may reflect that different factors are involved in the cerebral reperfusion process including the pattern of brain injury and regional-dependent perfusion response. It is possible that our finding of persistent hyperperfusion reflects the pattern of injury observed in our group (only 2/74 infants with mild HIE had basal ganglia thalami injury). In agreement with us, other researchers have hypothesized that the hyperperfusion response may take longer to decrease or normalize in milder watershed injuries^8^.

So far different studies have highlighted the potential of ASL in HIE. De Vis et al. found higher CBF values in basal ganglia and thalami in the neonates with moderate or severe HIE who later developed adverse neurodevelopmental outcomes^7^. More recently, O’Gorman Tuura et al. showed that increased perfusion in the basal ganglia correlates with cognitive development even in case of mild impairment^9^. Similarly, Wang et al. identified an association between increased basal ganglia perfusion and neonatal behavioural neurological assessment at 28–30 days of age in a cohort of forty neonates with different severity stages of HIE^11^. Our study adds to this research showing that elevated cerebral perfusion may be associated with long-term neurological outcomes in neonates with mild HIE. This is extremely important as neurodevelopmental impairments may become apparent only later in mild HIE. Therefore, early prediction of outcomes may improve identification of the high-risk infants who need a closer follow-up.

Although preclinical evidence suggests that thalamic injury may occur even after brief episodes of ischaemia^26^, only a small percentage of infants with mild HIE exhibit damage to the basal ganglia. In the PRIME study only 2 out of 52 infants (3.8%) with mild encephalopathy had moderate basal ganglia and thalamic changes^27^. In a retrospective analysis of 48 infants with mild HIE who were treated with therapeutic hypothermia, 2 (4%) had thalamic injury^5^. In a recent randomised controlled trail who recruited neonates with mild HIE, only 1 out of the 99 infants (1%) showed basal ganglia or thalami injury^28^.

Even though white matter injury is common in mild HIE, there is little evidence that white matter injury alone can predict later outcome. A recent study showed that white matter injury did not reach any significance to be included in the predictive model for death or neurodevelopmental impairment in HIE^29^. Another research revealed that white matter injury on conventional MRI had poor prognostic accuracy with a sensitivity of only 40% and a specificity of 69% to predict adverse outcome at 2 years^30^. These data underline that it is crucial to integrate conventional MRI with other imaging techniques to improve its diagnostic accuracy. This is confirmed by previous research, which suggested that thalamic metabolic perturbation and altered perfusion may occur despite the absence of apparent injury on conventional MRI in moderate and severe HIE. In a secondary analysis of the MARBLE study, MR spectroscopy abnormalities were common in the 92 infants with subcortical or periventricular white matter injury^30^. In the same way, Zheng et al. compared ASL perfusion between HIE neonates with and without conventional MRI abnormalities and found that CBF could successfully predict language and motor scores even without any apparent brain injury on conventional MRI^10^.

The severity of the hypoxic-ischemic insult may directly influence the brain areas affected, with distinct injury patterns based on HIE severity. Therefore, a better understanding of these patterns can have an impact on clinical outcomes and therapeutic approaches. In our study we found that infants with moderate and severe HIE had a significantly higher whole brain CBF than those with mild HIE after controlling for multiple confounding factors. This finding may reflect that an increasing duration and severity of the hypoxic-ischaemic insult may cause an increasing cerebral reperfusion. This is in keeping with preclinical models, which assume a linear relationship between the severity, duration of the hypoxic insult and the severity of injury, suggesting that mild encephalopathy represents the mild end of a spectrum. Preclinical models in fact, reported a significant injury to hippocampus even with a shorter duration of hypoxic injury, whereas a longer ischemia resulted in severe neuronal death in the parasagittal and lateral cortex, striatum, and most of the hippocampus^26^.

While there has been a significant improvement of the outcome after moderate and severe HIE, many infants go on to develop cognitive and memory deficits during childhood, even in the absence of cerebral palsy^31^. This is even more prominent in mild HIE, where increasing evidence suggests learning, behaviour abnormalities and increased risk of autism during childhood^3,32,33^. Our data suggest that multiple structures involved in memory and cognition such as hippocampus, parahippocampal gyrus, cingulate cortex and paracingulate gyri displayed higher CBF with increasing severity of HIE. These data highlight that by using ASL it is possible to assess cerebral perfusion into areas, which may be overlooked on neonatal MRI, but play a pivotal role as developmental trajectories in cognitive function.

This study has limitations. First the small sample size of infants with moderate and severe HIE may have affected the statistical power as far as the comparison across different HIE severity stages is concerned. Nonetheless, the substantial number of neonates with mild HIE strengthens the findings for this group. Future research should prioritize the validation of ASL as a biomarker for mild HIE, exploring its potential to improve prognostic accuracy. By integrating ASL with conventional MRI, clinicians could better identify infants who are at higher risk of adverse outcome later on. Second, there is no school-age assessment. Isolated white matter injury may have effects on later cognitive and behavioural outcomes. Therefore, longer-term follow-up studies are required. Third, in the mild HIE group some of the infants were cooled whereas others were not. Although our data did not show any difference in the CBF values between cooled and non-cooled infants and the analysis was adjusted for multiple factors including therapeutic hypothermia treatment and age at the time of MRI, residual confounding can still persist. Fourth, family socioeconomic status was not included in our analysis as this information was not available. The role of parental education and literacy environment on the cognitive outcomes of infants with HIE is increasingly recognised^34^. Therefore, future studies will have to include this information as part of the study design and analysis. Finally, increased cerebral blood perfusion depends on the severity as well as on the timing of the hypoxic ischaemic insult, which most of the times is unknown. Only serial MRI scans can help to better describe the trajectory of altered perfusion. However, given the mild severity of HIE in our cohort, a repeated MRI scan was considered unfeasible.

## Conclusions

This study shows that higher whole brain CBF after birth, particularly in the basal ganglia, was associated with worse neurodevelopmental outcomes in infants with mild HIE. Our results highlight that the severity of HIE is associated with distinct patterns of cerebral perfusion, affecting specific brain regions. Advanced imaging techniques such as ASL may be a potential biomarker, capable of identifying infants at risk of adverse outcomes even when conventional MRI appears normal. Future multicentre studies with larger cohorts and harmonized imaging protocols are needed to confirm these associations and determine their clinical utility. While our results support the use of ASL as a research tool to explore early brain perfusion dynamics in mild HIE, its clinical application as a prognostic biomarker remains to be validated. Near-Infrared Spectroscopy can be a valid adjunct because it is simple, albeit not as accurate as ASL, and can continuously monitor CBF at the cot side.

## Supplementary Information

Below is the link to the electronic supplementary material.

Supplementary Material 1

## Data Availability

Data are available upon reasonable and once all the different sub-studies have been published. Requests for the data should be addressed to P.M.
