# Supplementary material for: ASL reveals regional brain perfusion impairment in neonates with mild hypoxic ischemic encephalopathy
Source: Sci Rep. 2025 Aug 28;15:31676. doi: 10.1038/s41598-025-17246-0 (PMC12391493; doi:10.1038/s41598-025-17246-0)

Supplementary Table 1. List of all the regions of interest (in Italic) analysed.

**Region of interest**
Central region
Precentral gyrus
Postcentral gyrus
Rolandic Operculum
Frontal lobe
Lateral surface
Superior Frontal gyrus, dorsolateral
Middle Frontal gyrus
Inferior Frontal gyrus, opercular part
Inferior Frontal gyrus, triangular part
Medial surface
Superior Frontal gyrus, medial part
Supplementary motor area
Paracentral lobule
Orbital surface
Superior frontal gyrus, orbital part
Superior frontal gyrus, medial orbital
Middle Frontal gyrus, orbital part
Inferior Frontal gyrus, orbital part
Gyrus rectus
Olfactory cortex
Temporal lobe
Lateral surface
Superior temporal gyrus
Heschl gyrus
Middle temporal gyrus
Inferior temporal gyrus
Parietal lobe
Lateral surface
Superior Parietal gyrus
Inferior Parietal gyrus
Angular gyrus
Supramarginal gyrus
Medial surface
Precuneus
Occipital lobe
Lateral surface
Superior Occipital gyrus
Middle Occipital gyrus
Inferior Occipital gyrus
Medial and inferior surfaces
Cuneus
Calcarine fissure and surrounding cortex
Lingual gyrus
Fusiform gyrus
Limbic lobe
Temporal superior pole
Temporal middle pole
Anterior cingulate cortex and paracingulate gyri
Median cingulate cortex and paracingulate gyri
Posterior cingulate cortex
Hippocampus
Parahippocampal gyrus
Insula
Subcortical gray nuclei
Amygdala
Caudate nucleus
Lenticular nucleus, Putamen
Lenticular nucleus, Pallidum
Thalamus

Supplementary Table 2. Cerebral Blood Flow values in cortical and deep gray matter structures in cooled and non-cooled infants with mild encephalopathy.

**Brain region**[a]	**No therapeutic hypothermia**	**Therapeutic hypothermia**	**FDR**
CBF ml/100 g tissue/min			
Precentral gyrus	20.68 (8.73)	22.26 (10.56)	0.32
Superior Frontal gyrus, dorsolateral	19.57 (9.94)	20.82 (10.97)	0.32
Superior frontal gyrus, orbital part	17.36 (7.63)	18.89 (9.86)	0.36
Middle Frontal gyrus	17.62 (8.81)	19.11 (10.15)	0.32
Middle Frontal gyrus, orbital part	15.51 (7.63)	17.37 (9.81)	0.34
Inferior Frontal gyrus, opercular part	19.78 (7.16)	20.41 (9.10)	0.41
Inferior Frontal gyrus, triangular part	18.95 (7.05)	20.07 (8.77)	0.33
Inferior Frontal gyrus, orbital part	18.64 (6.94)	20.06 (9.32)	0.33
Rolandic Operculum	21.83 (6.50)	22.76 (8.76)	0.34
Olfactory cortex	23.52 (6.73)	24.54 (8.03)	0.35
Superior frontal gyrus, medial part	18.81 (7.73)	20.48 (9.36)	0.32
Superior frontal gyrus, medial orbital	18.85 (6.42)	20.77 (8.76)	0.36
Gyrus rectus	20.18 (6.84)	21.35 (9.48)	0.57
Insula	20.11 (6.65)	21.82 (8.92)	0.32
Anterior cingulate cortex and paracingulate gyri	20.14 (5.81)	21.64 (8.35)	0.32
Median cingulate cortex and paracingulate gyri	21.58 (5.84)	23.30 (8.36)	0.32
Posterior Cingulate cortex	18.79 (5.75)	20.22 (8.68)	0.32
Hippocampus	26.20 (6.58)	27.56 (8.20)	0.36
Parahippocampal gyrus	25.76 (6.67)	26.76 (7.95)	0.36
Cuneus	15.49 (5.74)	18.44 (8.63)	0.32
Amygdala	22.78 (7.14)	24.34 (8.20)	0.34
Supplementary motor area	24.22 (8.43)	25.19 (10.32)	0.34
Calcarine fissure and surrounding cortex	18.90 (5.73)	21.77 (9.26)	0.32
Lingual gyrus	20.83 (6.64)	23.56 (9.38)	0.32
Superior Occipital gyrus	13.82 (6.35)	16.36 (9.08)	0.32
Middle Occipital gyrus	14.81 (6.44)	17.74 (8.79)	0.32
Inferior Occipital gyrus	17.40 (7.42)	19.41 (9.56)	0.32
Fusiform gyrus	20.16 (7.64)	22.20 (9.58)	0.32
Postcentral gyrus	21.19 (8.56)	22.75 (10.24)	0.34

Superior Parietal gyrus	15.74 (8.96)	17.21 (10.32)	0.33
Inferior Parietal gyrus	16.84 (7.77)	18.44 (9.42)	0.32
Supramarginal gyrus	18.05 (7.36)	19.99 (9.04)	0.32
Angular gyrus	15.55 (6.73)	17.43 (8.68)	0.32
Precuneus	17.05 (6.63)	18.95 (8.57)	0.32
Paracentral lobule	23.80 (9.30)	25.16 (11.32)	0.36
Caudate nucleus	18.81 (6.31)	20.28 (8.01)	0.32
Lenticular nucleus, Putamen	23.97 (7.24)	25.63 (9.23)	0.32
Lenticular nucleus, Pallidum	20.98 (7.24)	24.28 (7.90)	0.32
Thalamus	24.94 (7.99)	27.81 (10.23)	0.32
Heschl gyrus	24.51 (7.50)	26.24 (9.54)	0.32
Superior temporal gyrus	20.32 (6.94)	21.83 (8.68)	0.32
Temporal superior pole	20.87 (7.47)	22.26 (9.47)	0.32
Middle temporal gyrus	17.54 (6.88)	19.53 (8.74)	0.32
Temporal middle pole	19.86 (8.63)	20.63 (10.73)	0.76
Inferior temporal gyrus	19.38 (8.94)	20.88 (10.60)	0.33

FDR, false discovery rate.

Supplementary Table 3. Cerebral Blood Flow values in cortical and deep gray matter structures in infants with mild hypoxic-ischaemic encephalopathy with normal and adverse outcome.

**Brain region**[a]	**Normal outcome**	**Adverse outcome**	**P value**[b]
CBF ml/100 g tissue/min			
Precentral gyrus	19.12 (8.98)	32.18 (9.12)	<0.001
Superior Frontal gyrus, dorsolateral	18.15 (10.01)	30.73 (7.94)	<0.001
Superior frontal gyrus, orbital part	17.15 (8.83)	28.03 (7.28)	<0.001
Middle Frontal gyrus	16.43 (9.09)	28.68 (7.48)	<0.001
Middle Frontal gyrus, orbital part	14.61 (8.44)	26.31 (7.11)	<0.001
Inferior Frontal gyrus, opercular part	18.18 (7.83)	29.91 (7.37)	<0.001
Inferior Frontal gyrus, triangular part	17.79 (7.58)	28.76 (7.18)	<0.001
Inferior Frontal gyrus, orbital part	17.52 (7.71)	29.03 (7.43)	<0.001
Rolandic Operculum	20.45 (7.20)	32.26 (9.64)	<0.001
Olfactory cortex	22.35 (7.15)	32.77 (7.46)	<0.001
Superior frontal gyrus, medial part	17.95 (8.24)	29.55 (7.44)	<0.001
Superior frontal gyrus, medial orbital	18 (6.87)	28.96 (7.89)	<0.001
Gyrus rectus	18.77 (7.57)	30.83 (7.95)	<0.001
Insula	18.96 (7.21)	31.17 (8.39)	<0.001
Anterior cingulate cortex and paracingulate gyri	19.30 (6.55)	30.65 (8.81)	<0.001
Median cingulate cortex and paracingulate gyri	20.69 (6.58)	31.64 (7.78)	<0.001
Posterior Cingulate cortex	17.69 (6.85)	28.74 (7.52)	<0.001
Hippocampus	25.34 (7.16)	35.99 (7.16)	<0.001
Parahippocampal gyrus	24.75 (7.18)	34.81 (6.46)	<0.001
Cuneus	15.26 (6·51)	26.78 (8.83)	<0.001
Amygdala	22 (8.02)	33.25 (7.62)	<0.001
Supplementary motor area	21.62 (7.9)	34.44 (8.84)	<0.001
Calcarine fissure and surrounding cortex	18.37 (6.90)	30.75 (8.26)	<0.001
Lingual gyrus	20.04 (7.27)	32.58 (7.21)	<0.001
Superior Occipital gyrus	13.31 (7.18)	24.52 (8.44)	<0.001
Middle Occipital gyrus	14.61 (7.22)	25.55 (7.71)	<0.001
Inferior Occipital gyrus	16.41 (8.09)	27.90 (7.38)	<0.001
Fusiform gyrus	19.22 (8.21)	30.94 (6.48)	<0.001
Postcentral gyrus	19.65 (8.79)	31.84 (8.62)	<0.001
Superior Parietal gyrus	14.20 (8.91)	25.64 (7.55)	<0.001

Inferior Parietal gyrus	15.68 (8.10)	26.41 (6.95)	<0.001
Supramarginal gyrus	17.33 (7.76)	28.01 (7.21)	<0.001
Angular gyrus	14.77 (7.38)	24.95 (6.30)	<0.001
Precuneus	16.05 (6.88)	26.92 (7.27)	<0.001
Paracentral lobule	21.70 (9.04)	34.47 (10.61)	<0.001
Caudate nucleus	17.75 (6.91)	27.89 (6.98)	<0.001
Lenticular nucleus, Putamen	22.70 (7.38)	35.85 (9.79)	<0.001
Lenticular nucleus, Pallidum	20.92 (7.38)	31.58 (6.90)	<0.001
Thalamus	24.18 (8.32)	37.28 (9.80)	<0.001
Heschl gyrus	23.67 (7.46)	36.59 (11.08)	<0.001
Superior temporal gyrus	19.44 (7.30)	30.84 (8.08)	<0.001
Temporal superior pole	19.85 (8.12)	31.62 (7.40)	<0.001
Middle temporal gyrus	16.97 (7.52)	27.63 (6.58)	<0.001
temporal middle pole	18.68 (8.93)	29.27 (9.28)	<0.001
Inferior temporal gyrus	18.10 (9.42)	30.22 (7.22)	<0.001

CBF Cerebral Blood Flow; *SD* standard deviation.

[a]Data are presented as mean (standard deviation). Bold indicates statistical significant of $p<0.05$. [b]P values for the differences of cerebral blood flow values between the two groups were determined for each region separately by using analysis of covariance (ANCOVA) while controlling the effect for birth gestational age, gender, postnatal age at MRI, haematocrit values and therapeutic hypothermia.

Supplementary Figure 1. Box plots of cerebral blood flow values (ml blood/100 g tissue/min) for the neonates with mild (blue) compared with moderate or severe (red) hypoxic ischaemic encephalopathy. Only the statistically significant regions identified by the multivariate analysis of variance (MANOVA) are shown. Medians are indicated by horizontal lines; boxes outline the upper and lower quartiles; and the whiskers indicate 1·5×IQR from upper and lower quartiles. Outliers are indicated with dots lying beyond the whiskers.

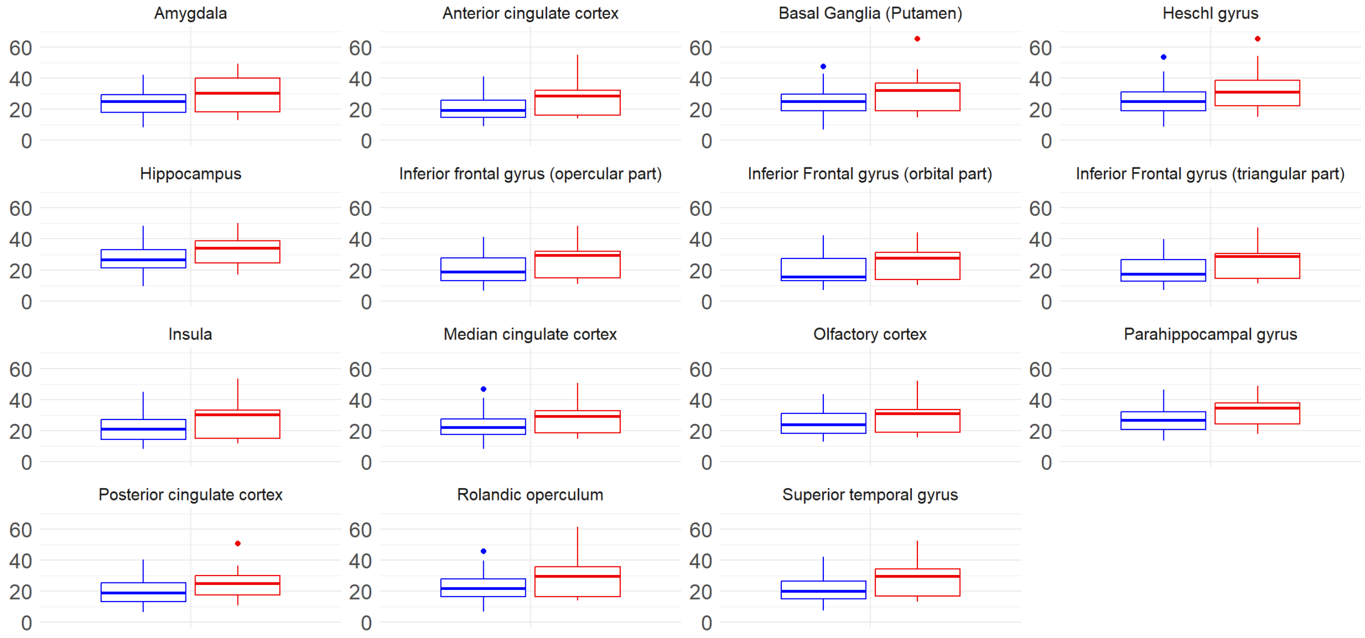

Supplement: Supplementary file 1 — Supplementary Material 1 [file 41598_2025_17246_MOESM1_ESM.pdf]
